# Circulating tumor cells: quintessential precision oncology presenting challenges for biology

**DOI:** 10.1038/s41698-017-0019-9

**Published:** 2017-05-08

**Authors:** Rebecca J. Morris

**Affiliations:** The Hormel Institute, University of Minnesota, Austin, USA

It is now well-known that cells from many types of malignant epithelial tumors delaminate and enter the circulation. These metastatic cells are known as circulating tumor cells or CTCs. CTCs have received wide attention because they offer the promise of a simple blood test to monitor the progress of cancer therapy, to aid in diagnosis and prognosis, and to define tumor evolution. Hence the liquid biopsy.^1^

CTCs are rare cells, as few as 1 in 10^9 in a typical sample of blood. Nevertheless, it is now technically possible to isolate and characterize them based on their molecular and genetic characteristics. For the most part, CTCs are rarely detected in the peripheral blood of healthy persons; however, CTCs in the blood of cancer patients reflect disseminated disease.^2^

Because of their epithelial nature, CTCs are usually detected first by depleting hematopoietic cells by CD45 (common leukocyte antigen) conjugated magnetic beads, followed by attaching fluorescence conjugated antibodies to the epithelial cell adhesion molecule, EpCAM, with further analysis for cytokeratins such as CK19, or by pan-cytokeratin imunoreactivity.^3^ Novel automated instrumentation such as the FDA cleared CellSearch System (Janssen Diagnostics, LLC, Raritan, NJ (formerly Veridex)) and the CytoTrack (CytoTrack ApS, Lyngby, Denmark) are used to collect and to analyze the cells.^4^ Other devices have been reported to aid isolation and characterization of CTCs. Even the downstream technology is promising, with the ability to perform reliably RNA-sequencing and DNA-sequencing on the single cells. Examples of several published instruments for CTC analysis with advantages and disadvantages are summarized in Table 1.

**Table 1:** Several examples of instruments in use for detection and analysis of CTCs

Instrument	Primary method for CTC detection	Secondary method for CTC detection	Advantages or disadvantages	Notes	Reference
CellSearch	Polymer-coated magnetic nanoparticles conjugated with EpCAM	Magnetic bead separation followed by immunofluorescence microscopy for cytokeratins and other markers	Advantage—93% CTC recovery, 1 cell/7.5 ml of blood Disadvantage—cells must be permeabilized	FDA cleared	Crowley et al.^3^
CytoTrack	Immunostaining cells on a “Cytodisc”	Scoring of “Cytodisc” by visual inspection	Visual inspection can be both an advantage and disadvantage	Gives similar results to the “CellSearch” device	Hillig et al.^10^
Screen Cell	Cell size: filtration of CTCs with the Screen Cell device	Captured cells are ready for cell culture, immunostaining or molecular biology	Advantage—CTCs are available for live cell in vitro methods	Potential for in vitro functional analysis	Desitter et al.^11^
Viatar CTC Solutions	Oncopheresis (dialysis) CTCs are the least deformable peripheral blood cells	Downstream detection of immunoreactive cells	Advantage—can collect more live cells than other methods Disadvantage—requires 4 h of machine dialysis	Undergoing human clinical trials, potential removal of metastatic cells	Coumans et al.^12^
Viator Technologies	Photoaccoustic detection	Detection and quantitation of CTCs	Advantage—works well for pigmented cells such as melanoma or with gold nanoparticle tagged CTCs	Selection is independent of immune-reactivity	Obrien et al.^13^
Micro-Hall detector	Micro fluidics together with immunomagentic detection of cancer stem cell therapeutic targets	Downstream profiling of single cells	Advantage—high throughput, high sensitivity	~ 50 times more sensitive than CellSearch	Issadore et al.^14^

However, despite the technological advances in identifying and characterizing CTCs, there are still significant biological challenges.

The first challenge quite simply, is sample size: as few as 1/10^9 cells per 7 ml sample of blood. Although the results of sequencing undeniably inform the nature of the cell in question, what does a single gene expression profile (or five or even ten), tell us about the biology a tumor or its metastases? Indeed EpCam expression can vary in CTCs, and amplification prior to sequencing RNA or DNA from single cells can induce bias. The former problem has been addressed by Sakurai et al.^5^ who targeted the CTCs a conditionally replicating adenovirus containing a green fluorescent protein (rAd-GFP) with human telomerase reverse transcriptase (hTERT). The rAd-GFP construct could thus proliferate in the hTERT-positive CTCs such that they expressed GFP. This aspect enabled an enrichment of CTCs regardless of EpCAM expression. With regard to the latter problem, it should also be possible to induce pluripotent stem cells from individual CTCs enabling clonal amplification of the cells and less bias upon sequencing. Moreover, the number of CTCs approximates the number of fetal cells persisting in the blood of female patients.^6^ Surely it is important to control for fetal chimerism in breast or ovarian cancer patients? Perhaps by inclusion of Y-Fluorescence In Situ Hybridization or other tissue-type markers?^7^

A second major challenge to biologists is insufficient knowledge of cytokeratin positive cells present in the blood of “healthy” individuals.^8^ Although often attributed to occult malignancy, there are other tantalizing biological questions waiting to be answered such as whether these rare cells might reflect an undiscovered function of normal epithelial cells, or even an undiscovered circulating epithelial progenitor. A related challenge is determining the significance of circulating epithelial cells in non-cancerous inflammatory disorders.^9^ Very little is known about this. Such knowledge could illuminate epithelial biology in general and inform inflammatory processes in particular. Moreover, we would conjecture that a comparison of the quantification of healthy-cell quantitative variable measures with corresponding disease-cell variable measures would also be informative.

A third challenge is the heterogeneity within the population of CTCs; how does one detect their possible transitions between epithelial and mesenchymal profiles. Here, additional markers for EMT such as twist, snail, and slug could be added.^8^ It is possible that mouse models of cellular lineage tracing could be applied to inform these cellular transitions during tumor evolution.

In summary, recent advances in detection and analysis of CTCs offer the promise of a liquid biopsy, laying the foundation for discovery of new tumor biomarkers and new knowledge on circulating epithelial cells in general. However, there are significant quantitative and biological challenges in this rapidly evolving field. Solutions to these challenges are as difficult as they are obvious, but are surely worth the effort.
